# Topical Simvastatin Enhances Tissue Regeneration in Full-Thickness Skin Wounds in Rat Models

**Published:** 2014

**Authors:** Mahsima Khoshneviszadeh, Soheil Ashkani-Esfahani, Mohammad Reza Namazi, Ali Noorafshan, Bita Geramizadeh, Ramin Miri

**Affiliations:** a *Medicinal and Natural Products Chemistry Research Center, Shiraz University of Medical Sciences, Shiraz, Iran.*; b *Student Research Committee, Shiraz University of Medical Sciences, Shiraz, Iran.*; c *Skin Research Center, Shiraz University of Medical Sciences, Shiraz, Iran.*; d *Histomorphometry and Stereology Research Centre, Shiraz University of Medical Sciences, Shiraz, Iran. *; e *Department of Pathology, Namazi Hospital, Shiraz University of Medical Sciences, Shiraz, Iran.*

**Keywords:** Simvastatin, Wound healing, Stereology, Reepithelization

## Abstract

Wounds and wound healing have always been one of the most important subjects that experimental researches were dedicated to. Simvastatin has been used for long as a common lipid lowering agent which was reported to have some pleiotropic effects such as antioxidation, anti-inflammation and immunomodulation. In this study we aimed to determine the effect of simvastatin on wound healing process in laboratory rats by means of stereological and histopathological analyses.

36 male Sprague-Dawley rats (220 ± 20 g) with a 1 cm^2^ circular full-thickness wound on their back were divided into three groups: SS group that received a gel with 2% concentration of simvastatin; UW group that received no treatment but daily irrigation with normal saline; Base group that was treated with the gel base. Duration of the study was 12 days and at the end, wound closure rate, grade of inflammation, granulation-tissue formation, ulceration, epithelization, fibroblast proliferation, collagen-bundles synthesis, and vascularization were determined.

Outcome of this study revealed that Simvastatin improves the wound healing by its anti-inflammatory and epithelization induction effect as well as statistically significant induction of fibroblast proliferation and collagen bundle synthesis which were reported by our stereological and histopathological investigations.

Results of the present study demonstrated that topical Simvastatin enhances the wound healing process through affecting different aspects of tissue regeneration; however, further researches are needed to find the exact mechanism, advantages and disadvantages of this chemical agent.

## Introduction

The wound healing process of skin involves several steps including angiogenesis, reepithelization, fibroblast activation and migration, and endothelial cell proliferation, all along with inflammatory response and oxidative reaction in the damaged tissue (1). The wound repair process begins immediately after injury by the release of different growth factors, cytokines, and *etc*. In addition, some complications such as disruption of blood vessels, infection and advanced inflammation might be occurred during the repair process (2).

Simvastatin (SS), a HMG-CoA reductase inhibitor, is one of the statin derivatives commonly used as lipid lowering agent. Moreover, different studies demonstrated the various properties of SS such as immunomodulation, anti-inflammation, endothelial function improvement, endothelial nitric oxide synthase inhibition, and anti sepsis effect. Different experimental studies suggested that pleotropic effects of SS are independent of its lipid lowering property. Recent studies also reported possible improving influence of SS in the wound healing process; however, the safety profile of SS was declared excellent especially in local treatments and major side effects were rarely observed and were mostly reversible (3-7).

Since SS presented positive effects on mechanisms that are contributed to wound healing promotion, in this study we aimed to determine the healing effect of this agent on full thickness skin wounds in laboratory rats. There was no report of using unbiased stereological methods (to calculate the rates of wound closure, fibroblast proliferation, collagen synthesis and vascularization) beside histopathological analyses for determining the effects of SS on full thickness skin wound.

## Experimental

*Preparation of SS gel*

SS was obtained from the Pharmacology Department of Shiraz University of Medical Sciences, Shiraz, Iran. In order to facilitate its application, we prepared 2% SS gel by dissolving 2 g SS in 2 cc ethanol (70%), and then transferring the solution into 2% carboxymethylcellulose (CMC) (2 g CMC dissolved in 98 cc distilled water). The base of gel was also supplied by the same method but without SS component.

*Animals and Excision of wound model*

In an experimental study, 36 male Sprague-Dawley rats (220 ± 20 g) of 2-3 months of age were chosen. General anesthesia was induced by intramuscular injection of mixture of Ketamine (Alfasan™, Woerden, Holland; 0.04 mL/100 g body weight) and Xylazine (Alfasan™, Woerden, Holland; 0.02 mL/10 g body weight). A 1 cm^2^ circular full-thickness wound was created on the posterior surface of animal’s neck. The rats were randomly divided into three groups. One group was treated with vehicle gel (Base group), a group treated with SS gel (SS group), and the untreated wounded group (UW group) that received no-treatment except cleaning of the wound surface with normal saline every day. After 12 days, the animals were euthanized with ether overdose and full thickness skin biopsies (1 cm×1 cm) were taken from the wound site and were fixed in buffered formaldehyde (pH = 7.2) for histopathological and stereological evaluations. 

The study protocol was approved by the Animal Ethics Committee of Shiraz University of Medical Sciences and the animal care was in accordance with their guidelines.

*Histopathological study*

All the specimens were fixed in formalin, paraffin blocks were provided from the specimens, and 15 µm slides were created from the skins and stained with Hematoxylin-Eosin. All the slides were inspected by a pathologist who was unaware of the groups. Scoring was done for acute and chronic inflammation, ulceration, granulation tissue formation and re-epithelization as presented in Table 1.

**Table 1:** Criteria used for light microscopy analysis

**Score**				**Criterion**
Chronic; Dominancy of chronic inflammatory cells in the field		Acute; Dominancy of neutrophils among all cells in the field		**Inflammation**
Present(1)		Absent(0)		**Ulceration**
Present(1)		Absent(0)		**Granulation tissue**
Full, covering 100%of the wound(3)	Moderate; covering > 50% of the wound(2)	Slight; covering < 50% of the wound(1)	Absent(0)	**Epithelization**

*Wound closure analysis*

**Figure 1:**
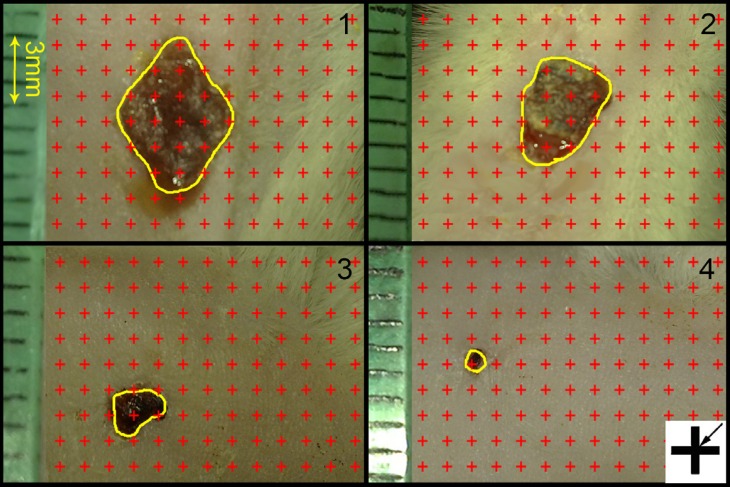
Digital photographs were captured from the wound surfaces every four days to measure the wound area. The total number of points within the wound borders (yellow line) was counted. As it is shown at the corner of this Figure, the right upper corner of the cross is considered as the point (arrow), and it is counted only if the right upper corner hits the wound surface. (1): day 1, (2): day 4; (3) day 8; (4) day 12 of Simvastatin-treated group

To determine the wound closure rate, images were captured from the wound surfaces every four days with a digital camera. To calibrate the magnification, a standard ruler was set at the level of the wound in each photograph, and the wound area at each visit was estimated by using a stereology software composed of a point grid (Figure 1) and by using the following formula: Area = ∑ P× a/p; where ∑P was the total points laid on the wound area and a/p, the area surrounded by every four crosses, was considered as the area per point (mm^2^) (8). Thereafter, the wound closure rate was calculated as: 

Wound closure rate (%) = ((area at visit 1 – area at each visit) / area at visit 1) ×100

*Tissue preparing and processing*

In a systematic random sampling manner (first with a random starting place and others with equal distances), nine pieces of the skin samples were cut and prepared for stereological analysis, each about 1 mm^2^. The pieces were embedded in a cylindrical paraffin block. The cylindrical blocks were sectioned using orientator methods for generating isotropic uniform random sections (9). The sections with 5 µm and 15 μm thickness were obtained and stained with both Hedenhain's azan and hematoxylin and eosin stains. Microscopic analyses of the dermis were done by using a video-microscopy system made up of a microscope (E-200, Nikon™, Japan) linked to a video camera, and a flat monitor. 

*Stereological analysis*

The volume densities of the collagen bundles (Vv; fraction of the unite volume of the dermis which is occupied by the collagen bundles) were estimated at final magnification of «×450» by using point counting method and the following formula (10, 11): 

*V*v_ (collagen/dermis)_ = P_ (collagen)_/P_ (dermis)_

**Figure 2:**
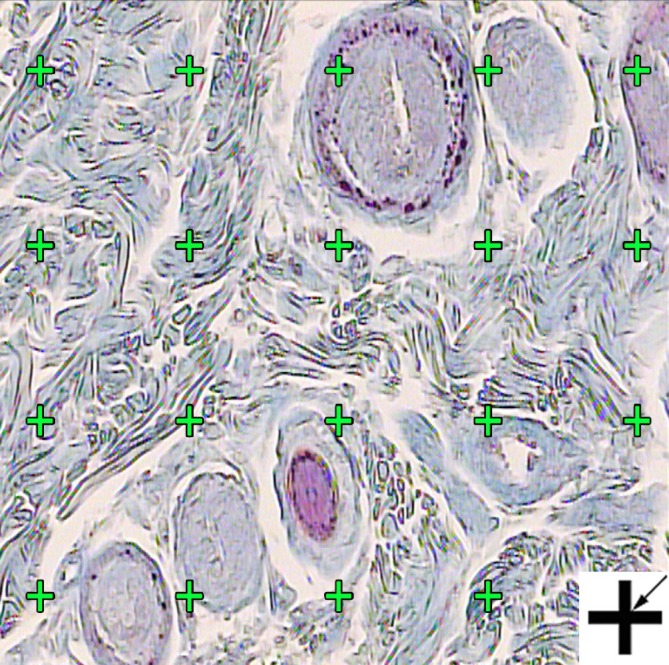
The volume density (Vv (collagen/dermis)) of the collagen fibers was estimated using a grid of points on the live image of dermis. The total number of points hitting the bundles is counted and divided by the total number of points hitting the reference space (dermis). A cross is presented at the corner of this figure. The cross is counted only if the right upper corner (arrow) hits the tissue. (Hedenhain's azan stain) (×450).

The P_ (collagen)_ was the number of points hitting the profiles of the collagen and P_(dermis)_ was the number of points hitting the dermis (Figure 2).

The length density of the vessels (Lv) and their mean diameter were estimated at final magnification of «×450» and by using the following formula (10, 11): 

L_v_ = 2×ΣQ/ ((a/f) ×Σf) 

**Figure 3:**
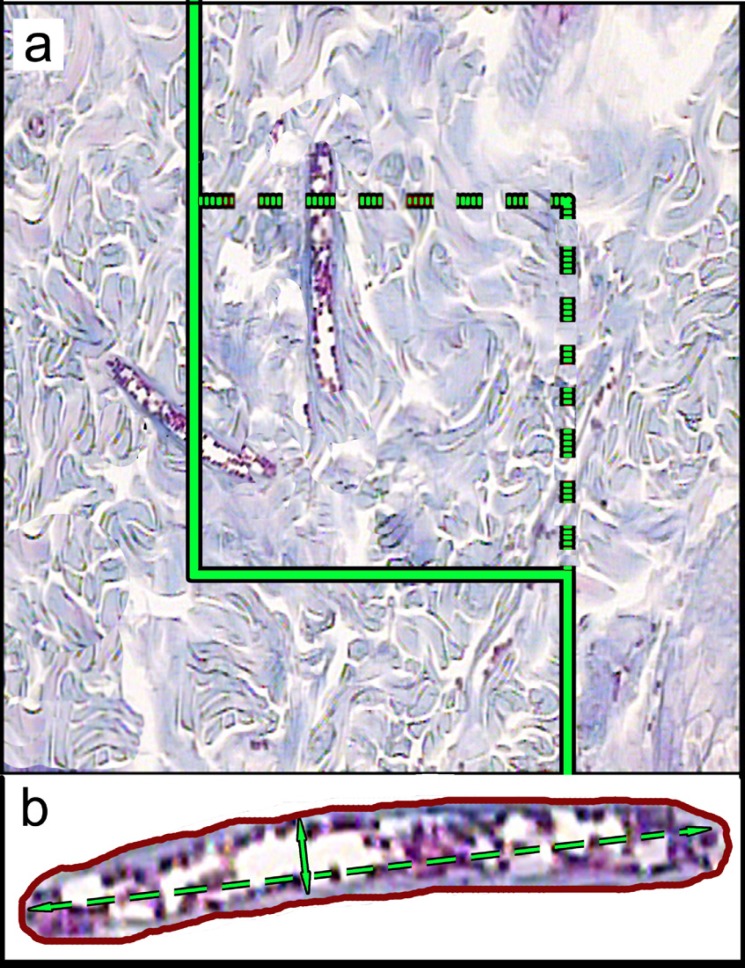
A: An unbiased counting frame is laid on the monitor image of wound dermis at final magnification of 450 randomly for estimation of the vessel’s length density (LV) and mean diameter. Any vessel lied in the counting frame or touched the inclusion borders (dotted lines) are selected. The vessels touched the exclusion borders (bold continuous lines), are omitted. B: Mean diameter of the vessel is estimated by measuring the short axis of the vessel (short double arrow). (Hedenhain’s azan stain

Where "∑*Q*" was the total number of vessel profiles counted per rat sample, (a/f) was the area of the counting frame, and "∑f" is the total number of frames counted per animal. Mean diameters of the vessels were calculated by measuring the short axis of a sampled vessel as its diameter (Figure 3). For these purposes, 5 µm sections were employed.

**Figure 4:**
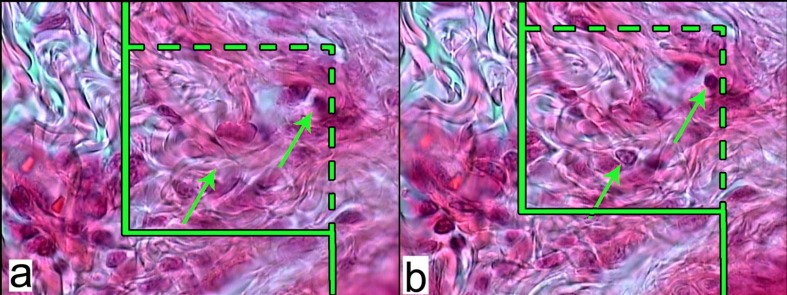
a: An unbiased counting frame laid on the image of the sections to estimate the numerical density (NV) of the fibroblasts. The nucleuses are unclear at the first 5 µm optical section (height of disector). b: As above, any nucleus lied in the counting frame or touched the inclusion borders (green dotted lines) and did not touch the exclusion borders (continuous green lines) and come into maximal focus within the next traveling 5 µm optical section (height of disector) are counted (the two green arrows). (Hedenhain’s azan stain ×2000).

The numerical density (Nv; number of the cells per unit volume of the dermis) of the fibroblasts was estimated by employing 15 μm sections at magnification of ×2000 (Figure 4), the “optical disector” method and the following formula (10, 11):

*Nv* = ΣQ/ΣA× h

Where "ΣQ" was the number of nuclei coming into focus in the dissector height, "ΣA" was the total area of the unbiased counting frame in all microscopic fields and "h" was the height of disector (5 μm here). 

*Statistical analysis of the data*

Data were collected, analyzed and reported as mean and standard deviation (mean ± SD). Statistical comparisons between stereological outcomes of groups were carried out by using SPSS software (ver. 16.0, Chicago, IL, USA). One-way analysis of variance (ANOVA) followed by Tukey’s post test were used to analyze the data. P ≤ 0.05 was considered as statistically significant.

## Results

*Pathological study results*

According to pathological comments, ulceration, acute inflammation, granulation tissue formation, and slight epithelization (score 1) were the dominant presentations in the UW group. In base group, acute inflammation was observed in the rats as the governing type of inflammation; ulceration, granulation tissue formation and slight epithelization (score 1) were also dominant in base group. Group SS had no sign of inflammation at the day 12 except one of the animals with acute inflammation; full epithelization (score 3) was seen in the rats except one of them with score 2 epithelization.

*Wound closure rate*

**Figure 5:**
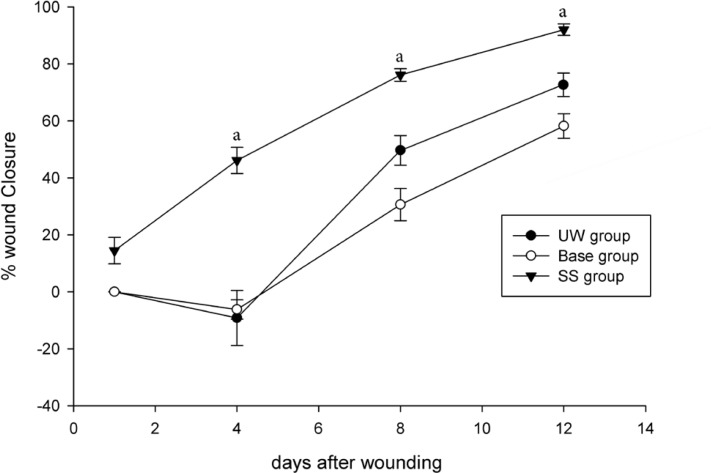
Effect of Simvastatin on wound closure rate in laboratory rats; untreated wounded (UW), gel base treated and SS-treated wounds in rats. Each point represents the mean (±SE) of twelve wounds. “a”: P<0.05, SS-treated group vs. UW and gel base treated groups

The initial area of the wounds was 104.37 ± 4.11 mm^2^ with insignificant difference among the experimental groups. The rate of wound closure in SS group was significantly higher in comparison with the UW and base groups (P < 0.05) (Figure 5). The base group was shown to have inconsequentially slower closure rate comparing to the UW group.

*Fibroblast population*

Numerical density of the fibroblasts (Nv) in the dermis of SS group was reported to be 103% higher than UW (P > 0.001) and 95% higher than the base group (P > 0.001) (Table 2).

*Volume density of the collagen bundles*

The volume densities of the collagen bundles were significantly higher in SS group comparing to those of the UW and base groups (P < 0.001) (Table 2). 

*Volume density, Length density and diameter of the vessels*

The mean of volume densities of vessels in SS group were insignificantly higher than UW and base groups by 26% (P = 0.79) and 52% (P = 0.36) respectively. Statistical analysis of length densities of the vessels revealed lower mean values in SS group (P = 0.79) and base group (P = 0.83) comparing to the UW group (Table 2).

**Table 2:** Mean (SD) of the numerical density of the fibroblasts (×10^4^ per mm^3^), volume densities of the collagen bundles (*V*v _collagen/dermis_; %) and vessels (*V*v _vessel/dermis_; %), length density (mm/mm^3^) and mean diameter (µm) of vessels in the dermis of the wounded rats treated with simvastatin gel (SS) , gel base (Base) and untreated wounded group (UW).

**Vessels**			**Collagen**	**Fibroblasts**	**Groups**
**Mean Diameter **	**Length density**	**% Volume density**	**% Volume density**	**Numerical density**	
2.20(0.44)	20.21(8.10)	3.25% (2.75%)	54 %( 4 %)	17.33(8.28)	**UW**
3.45(1.23)*	16.57(3.89)	4.09% (1.75%)	71% (6%)*	35.23(6.90)*	**SS**
1.22(0.28)	17.16(7.25)	2.40% (2.79%)	49% (5%)	18.02(6.65)	**Base**

* p < 0.05, SS group vs. UW group and Base group

Comparing the means of vessel diameters demonstrated that this parameter was higher than the base group by 57% (P = 0.01) in UW group and by 182% (P = 0.005) in SS group (Table 2).

## Results and Discussion

Cutaneous wound healing is a complex process involving inflammation, fibroblast proliferation, angiogenesis, collagen bundle formation, and finally tissue remodeling (1). The results of different studies demonstrated the wound healing properties of various exogenous substances and also instrumental therapies such as laser therapy which were in most cases beneficial on the healing process examined by experimental and clinical methods (12). 

Statins, particularly SS, are commonly used clinically for treatment of hypercholesterolemia. Moreover, various studies indicated different pleiotropic properties of statins such as anti-inflammatory, endothelium protective, anti-thrombotic and anti coagulant, immunomodulatory, and antioxidant activity (-, 13, 14). Previous experiments revealed that among different statin derivatives, SS was determined as one of the best anti-inflammatory agents (15, 16). In a study by Rego *et al*. it was shown that topical administration of SS on open skin wounds not only had anti-inflammatory effect, but also reduced bacterial infection of the wound site (17). Results of our study have suggested the anti-inflammatory effect of topical SS gel as well. Some studies exhibited that SS administration improved endothelial cell proliferation as well as neovascularization (18), which was also supported by outcomes of the present study. Cakmak *et al*. (2009) had shown that oral administration of SS improved intestinal wound healing in rats; they also mentioned the anti-oxidative and immunomodulatory impacts of this agent in their investigation (19).

In the present study, we showed that topical administration of 2% SS gel improved the rate of wound closure in laboratory rats. Pathological analyses also confirmed the anti-inflammatory effect of the agent as well as positive influence on granulation tissue formation and re-epithelization which hastened the healing process. Based on stereological outcomes, SS augmented the proliferation of fibroblasts, collagen bundles and also vessel formation which all accelerate the healing course; however, stereological parameters suggesting the vascularization induction influence were not statistically noticeable in contrast to other experimental groups.

To come into conclusion, SS revealed positive effect on the wound healing process considering pathological and stereological outcomes that were used in this experimental study; however, further investigations, particularly clinical studies, are needed to approve this agent as a treatment in skin injuries and wound healing and to determine the exact mechanisms involved.
